# Development of a machine learning model for predicting the expression of proteins associated with targeted therapy in endometrial cancer

**DOI:** 10.3389/fonc.2025.1502370

**Published:** 2026-01-12

**Authors:** Chenwen Sun, Qianling Li, Yanan Huang, Yang Xia, Meiping Li, Xiucong Zhu, Jinke Zhu, Zhenhua Zhao

**Affiliations:** 1 Department of Radiology, Shaoxing People’s Hospital, Shaoxing, China; 2 School of Medicine, Graduate School, Zhejiang University, Hangzhou, China; 3 Department of Radiology, Shaoxing Maternity and Child Health Care Center, Shaoxing, China

**Keywords:** endometrial carcinoma, machine learning, Pten, PIK3, mTOR, targeted therapy

## Abstract

**Background:**

To develop a machine learning model integrates multi-parametric magnetic resonance imaging (MRI) radiomics features and clinicopathological features to predict the expression status of phosphatase and tension homolog (PTEN), phosphatidylinositol-4,5-bisphosphate 3-kinase catalytic subunit alpha (PIK3CA), and mammalian target of rapamycin (mTOR), which are frequently linked with targeted therapy for endometrial cancer (EC), in order to establish a dependable foundation for personalized adjuvant therapy for EC patients.

**Methods:**

we retrospectively recruited 82 EC patients who underwent preoperative MRI and radical resection at two independent hospitals. 60 patients from Center 1 were utilized as the training set for constructing the machine learning model, while 22 patients from Center 2 served as an external validation set to assess the model’s performance. We evaluated the performance of models predicted three proteins’ expression using receiver operating characteristic (ROC) analysis, calibration curve analysis, and decision curve analysis (DCA).

**Result:**

To construct machine learning models for predicting the expression of PTEN, PIK3CA, and mTOR, we respectively screened 5 radiomic and 7 clinicopathologic features, 4 radiomic and 9 clinicopathologic features, and 2 radiomic and 10 clinicopathologic features. The area under the curve (AUC) values of the radscore, clinicopathology, and combination models predicting PTEN expression were 0.875, 0.703, and 0.891 in the training set, and 0.750, 0.844, and 0.833 in the validation set, respectively. The AUC values for the models predicted PIK3CA expression in the training set were 0.856, 0.633, and 0.880, respectively, in the validation set, they were 0.842, 0.667, and 0.825. The AUC of each model for mTOR were 0.896, 0.831, and 0.912 in the training set, and 0.729, 0.847, and 0.829 in the validation set. Calibration curve analysis and DCA showed that the combination models were both well calibrated and clinically useful.

**Conclusion:**

Machine learning models integrating multi-parametric MRI radiomics and clinicopathological features can be a potential tool for predicting PTEN, PIK3CA, and mTOR expression status in EC patients.

## Introduction

EC ranks among the most prevalent malignant tumors affecting the female reproductive system, and its incidence continues to rise each year (1). Although the majority of endometrial cancer patients can be diagnosed early and have a good prognosis after surgery, approximately 10% to 15% of advanced endometrial cancer patients still face limited treatment options and suboptimal systemic chemotherapy efficacy. The 5-year survival rate for patients with distant metastases is only 17% (2). Identifying risk factors for EC metastasis or recurrence remains challenging, and a reliable basis for individualized adjuvant therapy is lacking. Consequently, systemic toxicity often leads to overtreatment of EC. However, given the advancements in research on tumorigenesis and molecular pathway targets associated with EC development, personalized targeted therapy still holds significant promise at this stage.

Mutated genes and aberrant signaling pathways that induce the development of endometrial cancer vary. Notably, the PIK3/AKT/mTOR pathway exhibits the highest rate of alterations among solid tumors, particularly in 92% of type I and 60% of type II endometrial cancers. This is primarily attributed to the prevalence of mutations in genes such as PTEN and PIK3CA. Such findings underscore the significant role played by the PIK3/AKT/mTOR signaling pathway in the pathogenesis of endometrial cancer (3–5). The hyperactivation of the PIK3/AKT/mTOR pathway generally results from the loss of function of PTEN, the amplification or mutation of PIK3CA, and the elevated expression of the PIK3R1, AKT genes, and mTOR (3, 6). Therefore, targeting drugs against this pathway has become an increasing focus of scholarly attention.

MRI is widely used for the diagnosis of endometrial cancer due to its good soft tissue contrast. The International Federation of Gynecology and Obstetrics (FIGO) recommends MRI findings as the preferred staging basis for endometrial cancer. However, MRI-based imaging findings are susceptible to subjective factors due to their high inter-observer variability and lack of quantitative, objective assessment markers. Radiomics, a non-invasive and rapid method, quantitatively analyzes medical imaging data and transforms it into high-dimensional, mineable data. This enables the assessment of tumor heterogeneity, providing valuable information for tumor typing and grading, gene localization, early treatment, and prognosis assessment that cannot be identified by gross observation. Combining radiomics data with clinical data holds promise as an approach to improve clinical management (7).

The aim of this study was to evaluate the effectiveness of a machine learning model combining clinicopathological features and multi-parametric MRI-based radiomics features in predicting the expression of common endometrial cancer targeted therapy-related proteins PTEN, PIK3CA and mTOR, and to assist in clinical decision-making to optimize personalized treatment for EC patients.

## Methods

### Patient

We conducted a retrospective analysis on 60 endometrial cancer patients diagnosed at Center A between 2013 and 2022 and 22 endometrial cancer patients diagnosed at Center B between 2017 and 2022. None of these patients received any preoperative treatment. All participating institutions’ institutional review boards approved the retrospective study, and all boards waived the requirement for written informed permission from patients.

The following were the inclusion criteria for this study (1): all patients had a surgical pathology diagnosis of endometrial cancer (2); all patients had a preoperative standardized pelvic MRI; and (3) all eligible postoperative pathology specimens were stored in the tissue bank.

Strict exclusion criteria were used, including (1): radiotherapy, preoperative neoadjuvant chemotherapy, or any other intervention directed at the lesion site prior to MRI or surgical treatment (2); more than two weeks between the preoperative MR examination and the surgery (3); poor image quality due to artifacts or an incomplete MRI sequence as assessed by the treating radiologist; and (4) the lesion site was less than 1 centimeter in diameter at maximum or the tumor border could not be clearly delineated.

### MRI acquisition

Magnetic resonance images were acquired using a Siemens Verio 3.0 T MR scanner (Germany) in center A and a 1.5T MRI scanner (Siemens Avanto, Germany) in center B. All patients were required to breathe freely in the supine position during data acquisition. The following sequences were acquired: the sagittal T2 weighing imaging (T2WI), the sagittal contrast-enhanced T1-weighted images (CE-T1WI), and axial diffusion weighing imaging (DWI) with apparent diffusion coefficient (ADC) maps with b-values of 0 and 800 s/mm2. The primary scanning sequence for Center A includes T2WI (TR/TE: 3480 ms/103 ms, FOV: 320×320 mm); CE-T1WI (TR/TE: 6700 ms, FOV: 288×288 mm); DWI (TR/TE: 6700 ms/92 ms, FOV: 160×160 mm); and ADC (TR/TE: 6700 ms/92 ms, FOV: 160×160 mm). The main scanning sequence for Center B includes T2WI (TR/TE: 5210 ms/79 ms, FOV: 320×320 mm); CE-T1WI (TR/TE: 735 ms/11 ms, FOV: 320×320 mm); DWI (8900 ms/84 ms, 160 mm×136mm); ADC (TR/TE: 8900 ms/84 ms, FOV:160 mm×136 mm).

### Image segmentation and feature extraction

**Figure 1:**
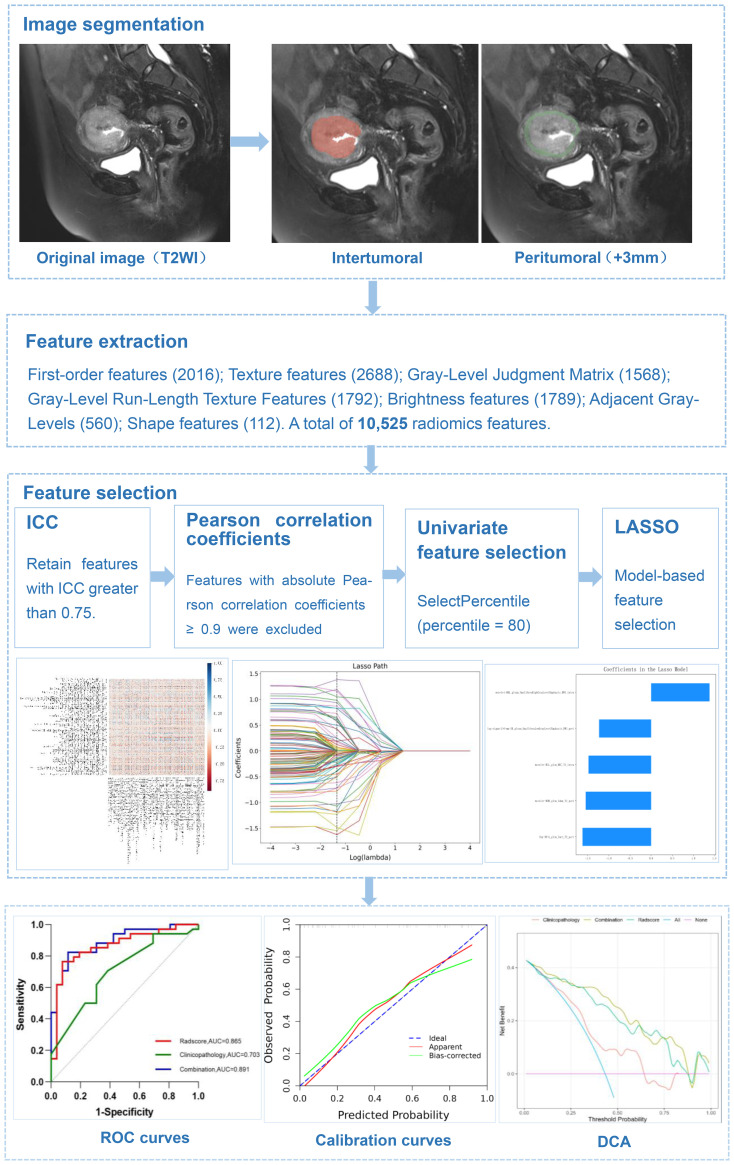
A diagram depicting overview of radiomics workflows.

A radiologist(reader 1)with 7 years of experience in gynecologic imaging manually delineated the tumor on four sequences (including T2WI, CE-T1WI, DWI, and ADC) using 3D slicer software (5.2.1) to obtain a volume of interest (VOI). The radiologist was not given any clinical or pathologic information about the patient. Following that, a VOI + 3 mm was created by increasing the volume of interest by 3 mm and manually deleting these spots (which included leiomyosarcomas, cysts, and effusions). The Artificial Intelligent Kit (AK) software (3.2.0.R, GE Healthcare, China) was used to extract imaging features from the VOI. (**Figure 1**).

A week later, reader 2 and reader 3 who have more than 5 years of experience with pelvic magnetic resonance imaging randomly selected 30 patients and performed the picture segmentation process. The intraclass correlation coefficient (ICC) was used to assess the consistency and reproducibility of radiomic features. Features with ICC greater than 0.75 were retained for further analysis.

### Pathological examination

**Figure 2:**
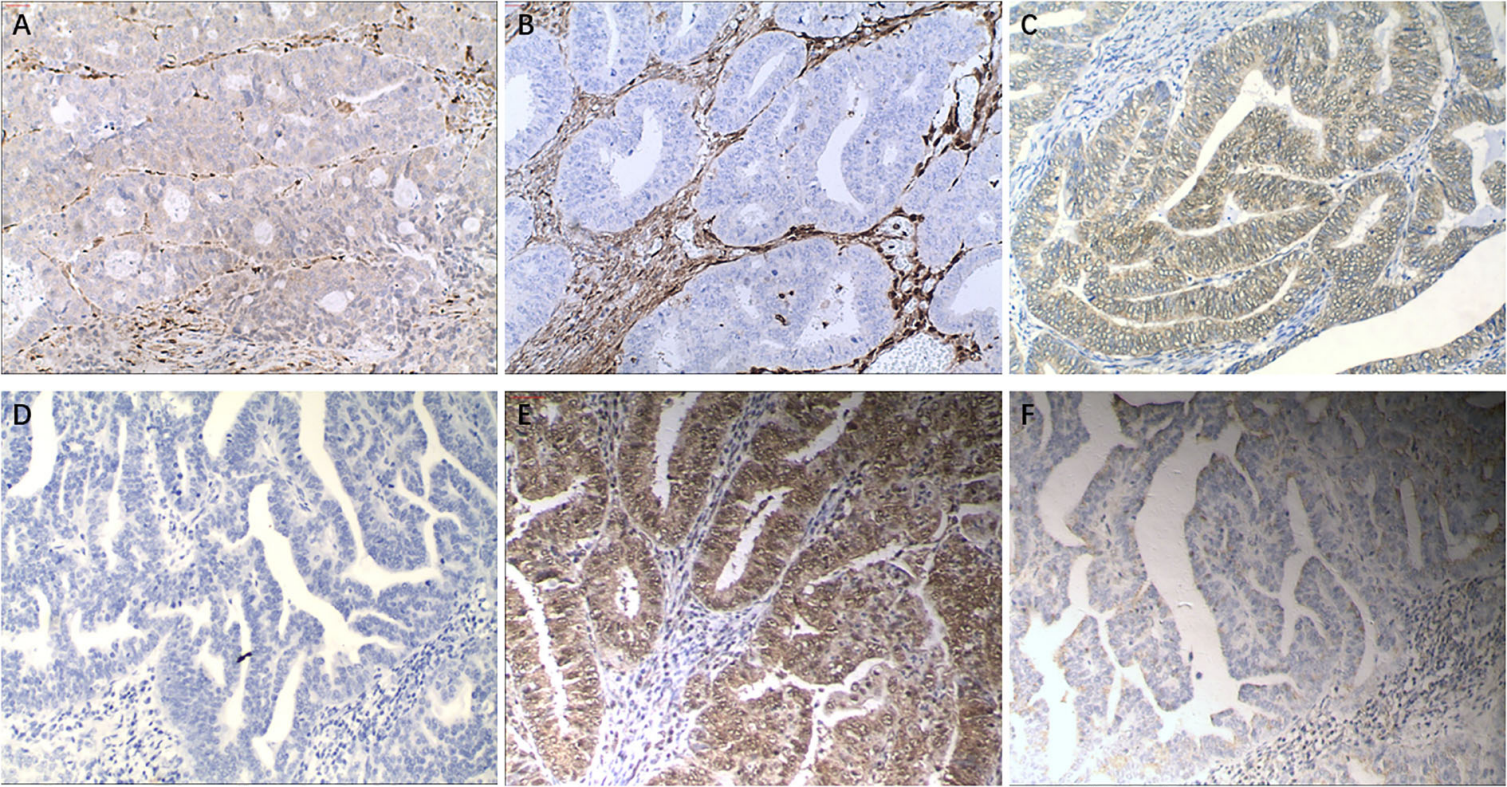
IHC images: Magnification: 10×40 (a, b): IHC images of PTEN; **(a)**, IRS: 8; **(b)**, IRS: 0; (c, d): IHC images of PIK3CA; **(c)**, IRS: 6; **(d)**, IRS: 1; **(e, f)**: IHC images of mTOR; **(e)**, IRS: 8; **(f)**, IRS: 2.

PTEN, PIK3CA and mTOR were assessed by immunohistochemistry (IHC) protocol using paraffin‐embedded tissue samples which were obtained from surgery. We used monoclonal mouse anti‐human PTEN antibody (RTU, RM265, GeneTech Co., Ltd., Shanghai, China), monoclonal rabbit antihuman PIK3CA antibody (1:100, SP139 Abcam, Shanghai, China), or monoclonal rabbit anti-human mTOR antibody (1:400, Y391, Abcam, Shanghai, China). Two professional pathologists with more than 8 years of diagnostic experience will perform the interpretation of the testing results. The immunostaining results of specific antibodies were measured semi-quantitatively by immunoreactive score (IRS) method. The specific calculation method of IRS is as follows: Staining intensity (SI) classification: 0, no staining; 1, light yellow; 2, brown yellow, 3, dark brown; percentage of stained cells (PP): 0, no staining; 1, staining in<10% of tumor cells; 2, staining in 10–50%; 3, staining in 50–80%; 4, staining in>80%. IRS=SI×PP. IRS > 3 was defined as positive immunoreactivity according to the literature criteria (8). An additional movie file shows this in more detail [see Additional file 1]. (**Figure 2**).

### Radiomics feature selection and modeling

First, radiomic features from the training set were standardized using the Z-score method to eliminate scale differences. To reduce feature redundancy, highly correlated features with an absolute Pearson correlation coefficient ≥ 0.9 were removed. Subsequently, dimensionality reduction and feature selection were performed using SelectPercentile (percentile = 80) and LASSO algorithm to further eliminate irrelevant features and retain radiomic features with strong predictive value. Meanwhile, for the collected 11 key clinicopathological features, the SelectKBest feature selection method was used to evaluate the relationship between each feature and the target variable, screening out features with stronger associations for the predictive model. The finally selected radiomic features and clinicopathological features were combined, and three different predictive models were constructed using logistic regression algorithm: a clinicopathological model, a radiomics score model, and a combined model. The discriminative performance of the three established models was evaluated through ROC analysis and quantified using corresponding AUC values with 95% confidence intervals (CI), sensitivity, specificity, and accuracy. Then, calibration curves were used to assess the consistency between the model-predicted probabilities and actual probabilities, and the clinical applicability of the models was evaluated through Decision Curve Analysis (DCA).

Meanwhile, in order to further explore whether the intra-tumor combined peri-tumor imaging model incorporating the 3mm region around the tumor is superior to the single intra-tumor or peri-tumor model, we constructed the intra-tumor combined peri-tumor imaging model based on the same method of feature screening and dimensionality reduction, retained the optimal features incorporated into the model, and built the single intra-tumor and single peri-tumor imaging models by single-factor and multifactorial logistic regression, respectively, and analyzed the predictive efficacy of the models by using the subjects’ ROCs and calculated AUCs, and the Delong test was used to compare the predictive performances of each model.

### Statistics

Data were statistically analyzed by applying SPSS 27.0 and R language (v.4.3.1) software. Differences in categorical indicators between risk groups were compared using the chi-square test or Fishers’ exact test, and differences in continuous variables were compared using the independent samples t-test or Mann-Whitney U-test, depending on whether they were normally distributed or not. In all two-sided tests, a P value <0.05 was considered statistically significant.

## Result

### Patient clinical characteristics

**Figure 3:**
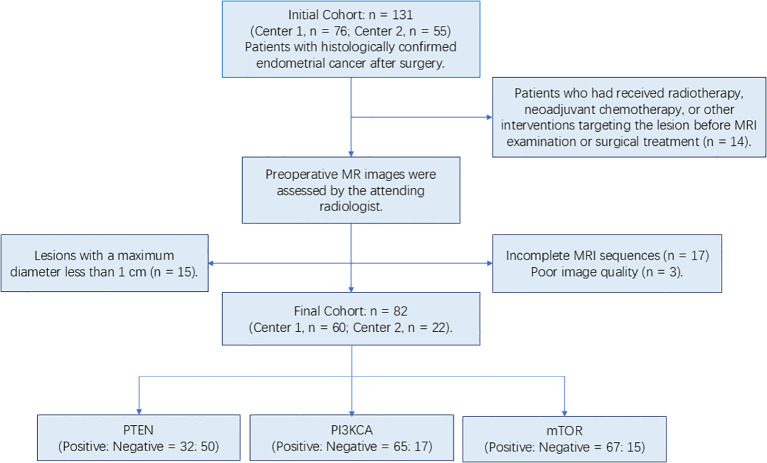
The workflow diagram for the inclusion and exclusion of the final cases.

This study initially comprised 76 patients from Center 1 and 55 patients from Center 2. Following the application of the exclusion criteria, a total of 82 patients were enrolled, with 60 from Center 1 and the remaining 22 from Center 2 (**Figure 3**). The mean age of the patients in the training group was 58.9 ± 2.47 (range: 34–77 years), and the mean age of the patients in the test group was 59.27 ± 4.26(range: 36–81 years). As shown in **Table 1**, no significant differences were found between the train group and the test group in terms of clinicopathologic indicators such as age, FIGO, myofibrillar infiltration, Ki-67 expression, and P53 expression (all P>0.05). However, in terms of Grade, a statistical difference was found between the train and test groups (P<0.05).

**Table 1:** Clinical parameters from the training and test groups.

Characteristics	Training cohort (%)	Test cohort (%)	p value
Total	60 (73.2)	22 (26.8)	
Age	58.9 ± 2.47	59.27 ± 4.26	0.876
FIGO			
Ia-Ib	52 (63.4)	19 (23.2)	0.972
II-III	8 (9.8)	3 (3.7)	
Grade			
I	39 (47.6)	6 (7.3)	0.002*
II-III	21 (25.6)	16 (19.5)	
Myometrial invasion			
Shallow myometrium	46 (56.1)	18 (22.0)	0.618
Deep myometrium	14 (17.1)	4 (4.9)	
Ki-67			
<30%;	10 (12.2)	3 (3.7)	0.166
30%-80%	47 (57.3)	15 (18.3)	
≥80%	3 (3.7)	4 (4.9)	
P53			
–	36 (43.9)	17 (20.7)	0.147
+	24 (29.3)	5 (6.1)	
ER			
–	9 (11.0)	3 (3.7)	0.877
+	51 (62.2)	19 (23.2)	
PR			
–	9 (11.0)	4 (4.9)	0.727
+	51 (62.2)	18 (22.0)	
Lymphatic node transfer			
–	57 (69.5)	21 (25.6)	0.933
+	3 (3.7)	1 (1.2)	
Vessel carcinoma embolus			
–	51 (62.2)	19 (23.2)	0.877
+	9 (11.0)	3 (3.7)	
Ovarian metastasis			
–	58 (70.7)	22 (26.8)	0.386
+	2 (2.4)	0 (0.0)	

Continuous variables were presented as mean ± standard deviation, while categorical variables were reported as patient numbers with percentages in parentheses. FIGO, International Federation of Gynecology and Obstetrics. *p<0.05, significant.

PTEN, PIK3CA, mTOR expression status and clinicopathological features are shown in **Table 2**. There were significant differences in terms of PTEN expression and Ki-67 expression, mTOR expression and EC Grade (p<0.05), and there were no significant differences in other clinicopathological features and protein expression status (p>0.05).

**Table 2A:** Clinical parameters of EC patients in the PTEN expression group and inexpression group.

	PTEN expression N (%)	PTEN inexpression N (%)	P value
Total	32 (39.0)	50 (61.0)	
Age	60.08 ± 9.98	57.31 ± 8.65	0.310
FIGO			
Ia-Ib	29 (35.4)	42 (51.2)	0.391
II-III	3 (3.7)	8 (9.8)	
Grade			
I	20 (24.4)	25 (30.5)	0.267
II-III	12 (14.6)	25 (30.5)	
Myometrial invasion			
Shallow myometrium	24 (29.3)	40 (48.8)	0.594
Deep myometrium	8 (9.8)	10 (12.2)	
Ki-67			
<30%	9 (11.0)	4 (4.9)	0.045*
30%-80%	21 (25.6)	41 (50.0)	
≥80%	2 (2.4)	5 (6.1)	
P53			
–	17 (20.7)	36 (43.9)	0.081
+	15 (18.3)	14 (17.1)	
ER			
–	5 (6.1)	8 (9.8)	0.964
+	27 (32.9)	42 (51.2)	
PR			
–	5 (6.1)	8 (9.8)	0.964
+	27 (32.9)	42 (51.2)	
Lymphatic node transfer			
–	31 (37.8)	47 (57.3)	0.555
+	1 (1.2)	3 (3.7)	
Vessel carcinoma embolus			
–	27 (32.9)	43 (52.4)	0.839
+	5 (6.1)	7 (8.5)	
Ovarian metastasis			
–	32 (39.0)	48 (58.5)	0.252
+	0 (0.0)	2 (2.4)	

*p<0.05, significant.

**Table 2B:** Clinical parameters of EC patients in the PIK3CA expression group and inexpression group.

	PIK3-CA expression N (%)	PIK3-CA inexpression N (%)	p value
Total	65 (79.3)	17 (20.7)	
Age	59.45 ± 2.34	57.29 ± 5.12	0.410
FIGO			
Ia-Ib	56 (68.3)	15 (18.3)	0.823
II-III	9 (11.0)	2 (2.4)	
Grade			
I	33 (40.2)	12 (14.6)	0.144
II-III	32 (39.0)	5 (6.1)	
Myometrial invasion			
Shallow myometrium	51 (62.2)	13 (15.9)	0.860
Deep myometrium	14 (17.1)	4 (4.9)	
Ki-67			
<30%	11 (13.4)	2 (2.4)	0.766
30%-80%	48 (58.5)	14 (17.1)	
≥80%	6 (7.3)	1 (1.2)	
P53			
–	42 (51.2)	11 (13.4)	0.994
+	23 (28.0)	6 (7.3)	
ER			
–	10 (12.2)	3 (3.7)	0.820
+	55 (67.1)	14 (17.1)	
PR			
–	10 (12.2)	3 (3.7)	0.820
+	55 (67.1)	14 (17.1)	
Lymphatic node transfer			
–	61 (74.4)	17 (20.7)	0.294
+	4 (4.9)	0 (0.0)	
Vessel carcinoma embolus			
–	54 (65.9)	16 (19.5)	0.252
+	11 (13.4)	1 (1.2)	
Ovarian metastasis			
–	64 (78.0)	16 (19.5)	0.301
+	1 (1.2)	1 (1.2)	

**Table 2C:** Clinical parameters of EC patients in the mTOR expression group and inexpression group.

	mTOR expression N (%)	mTOR inexpression N (%)	P value
Total	67 (81.7)	15 (18.3)	
Age	59.22 ± 9.44	58.00 ± 10.20	0.656
FIGO			
Ia-Ib	57 (69.5)	14 (17.1)	0.396
II-III	10 (12.2)	1 (1.2)	
Grade			
I	32 (39.0)	13 (15.9)	0.006*
II-III	35 (42.7)	2 (2.4)	
Myometrial invasion			
Shallow myometrium	51 (62.2)	13 (15.9)	0.372
Deep myometrium	16 (19.5)	2 (2.4)	
Ki-67			
<30%	8 (9.8)	5 (6.1)	0.122
30%-80%	53 (64.6)	9 (11.0)	
≥80%	6 (7.3)	1 (1.2)	
P53			
–	44 (53.7)	9 (11.0)	0.678
+	23 (28.0)	6 (7.3)	
ER			
–	12 (14.6)	1 (1.2)	0.281
+	55 (67.1)	14 (17.1)	
PR			
–	12 (14.6)	1 (1.2)	0.281
+	55 (67.1)	14 (17.1)	
Lymphatic node transfer			
–	63 (76.8)	15 (18.3)	0.332
+	4 (4.9)	0 (0.0)	
Vessel carcinoma embolus			
–	57 (69.5)	13 (15.9)	0.875
+	10 (12.2)	2 (2.4)	
Ovarian metastasis			
–	66 (80.5)	14 (17.1)	0.240
+	1 (1.2)	1 (1.2)	

*p<0.05, significant.

### Radiomics and clinical pathological feature selection

A total of 10,525 features were extracted from seven categories of 12 image preprocessing, including first-order features, shape features, texture features, luminance features (GLSZM), grayscale judgment matrix (GLDM), neighboring grayscale levels (NGTDM), and grayscale stroke length texture features (GLRLM).

Among the 10525 radiological features extracted from the MRI images, 9304 features were considered stable (ICC ≥ 0.75). First, feature pre-selection was performed by removing features with a Pearson correlation coefficient ≥ 0.9. Next, univariate feature selection was performed using SelectPercentile (percentile = 80), and finally, the most valuable radiological features were selected from the training cohort using the Lasso algorithm.

This study incorporated 7 clinicopathologic features associated with PTEN (FIGO, Myometrial invasion, Ki-67, P53, Lymphatic node transfer, Vessel carcinoma embolus, Ovarian metastasis) and 5 MRI-based radiomic features. Additionally, 9 clinicopathologic features related to PIK3CA (Age, Ovarian metastasis, Myometrial invasion, Ki-67, P53, ER, PR, Lymphatic node transfer, Vessel carcinoma embolus) along with 4 MRI-based radiomics features were selected to establish the models. Moreover, 10 clinicopathologic features associated with mTOR (Age, Vessel carcinoma embolus, Ovarian metastasis, FIGO, Grade, Myometrial invasion, Ki-67, P53, ER, Lymphatic node transfer) and 2 MRI-based radiomic features were utilized in model construction.

Based on the extracted radiomics features, we calculated a Radscore value for each patient, and then constructed a logistic regression model. **Table 3** shows the selected radiomics features. The Radscore calculation formulas related to the three protein expressions are shown as follows:

**Table 3:** Features for radiomics model construction.

	Feature name	Sequence	Region
PTEN	Lbp-3D-k_glcm_Imc1	T2WI	Peritumoral
	Log-sigma-2-0-mm-3D_glszm_SmallAreaLowGrayLevelEmphasis	DWI_	Peritumoral
	Wavelet-HHH_glcm_Idmn	T1WI	Peritumoral
	Wavelet-HHL_glszm_SmallAreaHighGrayLevelEmphasis	DWI	Intratumoral
	Wavelet-HLL_glcm_MCC	T1WI	Intratumoral
PIK3CA	Lbp-3D-k_firstorder_Minimum	ADC	Intratumoral
	Lbp-3D-k_glcm_Imc1	T2WI	Intratumoral
	Wavelet-HLL_glcm_ClusterShade	T2WI	Intratumoral
	Wavelet-LHL_firstorder_Skewness	T1WI	Intratumoral
mTOR	Log-sigma-2-0-mm-3D_glcm_Imc1	T1WI	Peritumoral
	Original_glszm_LargeAreaEmphasis	T2WI	Peritumoral
	Wavelet-HHH_glszm_HighGrayLevelZoneEmphasis	DWI	Peritumoral


$$\begin{array}{c} \text{PTEN}:\text{Radscore}=\;\\ -1.624\;\times \text{ Lbp}-3\text{D}-\text{k}\_\text{glcm}\_\text{Imc}1\_\text{T}2\_\text{peri }-\;1.232\;\\ \times \text{ Log}-\text{sigma}-2-0-\text{mm}\\ -3\text{D}\_\text{glszm}\_\text{SmallAreaLowGrayLevelEmphasis}\_\text{DWI}\_\text{peri }\\ -\;1.549\;\times \text{ Wavelet}-\text{HHH}\_\text{glcm}\_\text{Idmn}\_\text{T}1\_\text{peri }\\ +\;1.385\;\times \text{ Wavelet}\\ -\text{HHL}\_\text{glszm}\_\text{SmallAreaHighGrayLevelEmphasis}\_\text{DWI}\_\text{intra }\\ -\;1.483\;\times \text{ Wavelet}-\text{HLL}\_\text{glcm}\_\text{MCC}\_\text{T}1\_\text{intra} \end{array}$$

$$\begin{array}{c} PIK3CA:\text{Radscore}\\ =\;0.851\;\times \text{ Lbp}-3\text{D}-\text{k}\_\text{firstorder}\_\text{Minimum}\_\text{ADC}\_\text{intra }\\ +\;0.967\;\times \text{ Lbp}-3\text{D}-\text{k}\_\text{glcm}\_\text{Imc}1\_\text{T}2\_\text{intra }+\;0.944\;\\ \times \text{ Wavelet}-\text{HLL}\_\text{glcm}\_\text{ClusterShade}\_\text{T}2\_\text{intra }+\;1.599\\ \;\times \text{ Wavelet}-\text{LHL}\_\text{firstorder}\_\text{Skewness}\_\text{T}1\_\text{intra} \end{array}$$

$$\begin{array}{c} \text{mTOR}:\text{Radscore}\\ =-0.765\;\times \text{ Log}-\text{sigma}-2-0-\text{mm}\\ -3\text{D}\_\text{glcm}\_\text{Imc}1\_\text{T}1\_\text{peri }-\;1.050\;\\ \times \text{ Original}\_\text{glszm}\_\text{LargeAreaEmphasis}\_\text{T}2\_\text{peri }+\;0.809\\ \;\times \text{ Wavelet}\\ -\text{HHH}\_\text{glszm}\_\text{HighGrayLevelZoneEmphasis}\_\text{DWI}\_\text{peri} \end{array}$$

In addition, to evaluate the predictive performance of the intratumoral and peritumoral radiomics models separately, the intratumoral and peritumoral regions of each case were analyzed using the Artificial Intelligence Kit software to extract 5264 intratumoral radiomics features and 5261 peritumoral radiomics features, respectively. Through intraclass and interclass consistency tests, 4652 stable features (ICC ≥ 0.75) were selected from each region. The most valuable radiomics features were then chosen from the training cohort using the same selection method for model construction.

### Construction and evaluation of the model

**Figure 4:**
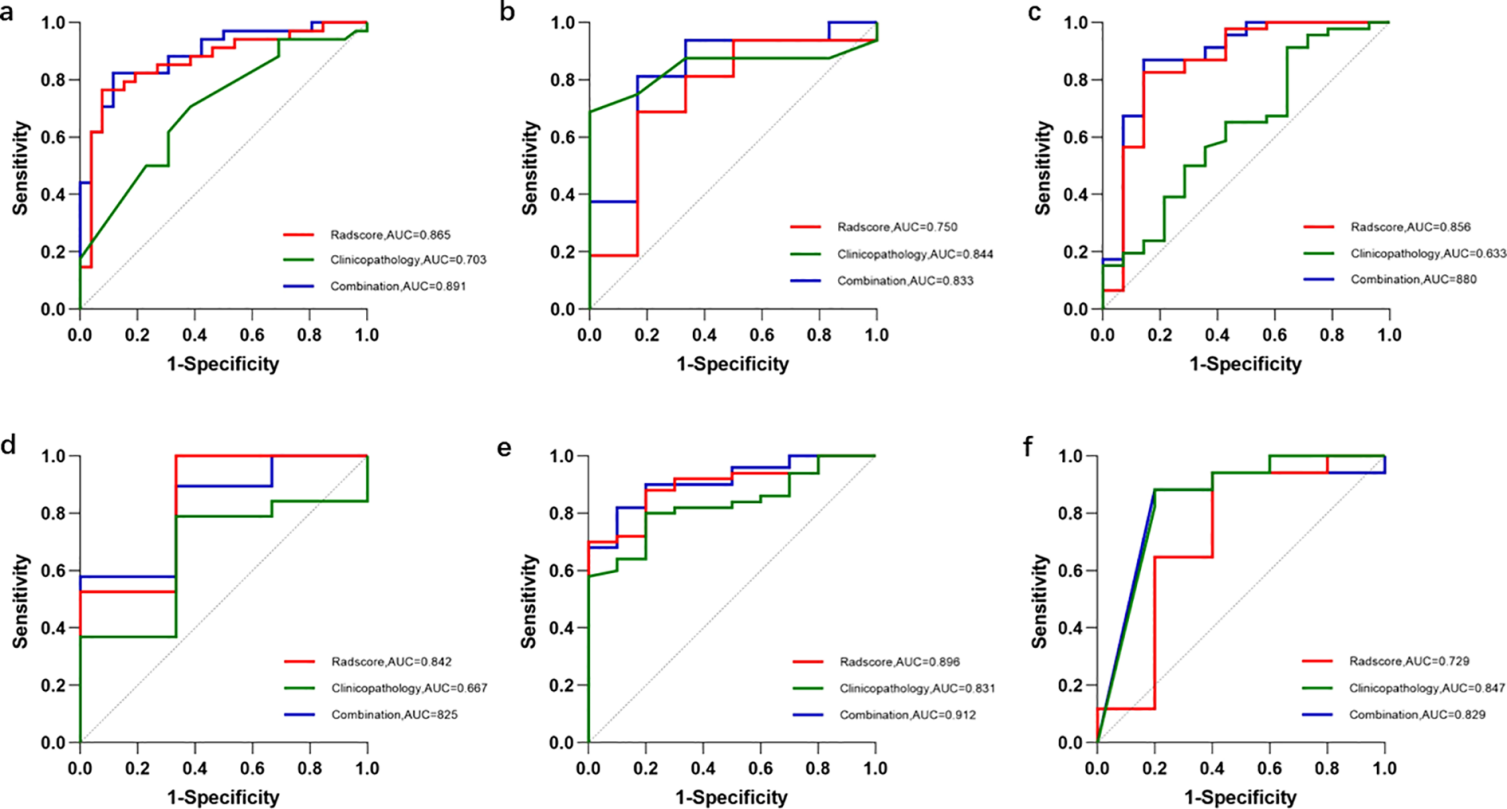
The ROC curves of the Radscore, Clinicopathology, and Combination model for each protein expression in the training and validation sets. **(a)** The ROC curves to predict PTEN expression status in the training set. **(b)** The ROC curves to predict PTEN expression status in the validation set. **(c)** The ROC curves to predict PIK3CA expression status in the training set. **(d)** The ROC curves to predict PIK3CA expression status in the validation set. **(e)** The ROC curves to predict mTOR expression status in the training set. **(f)** The ROC curves to predict mTOR expression status in the validation set.

**Figure 5:**
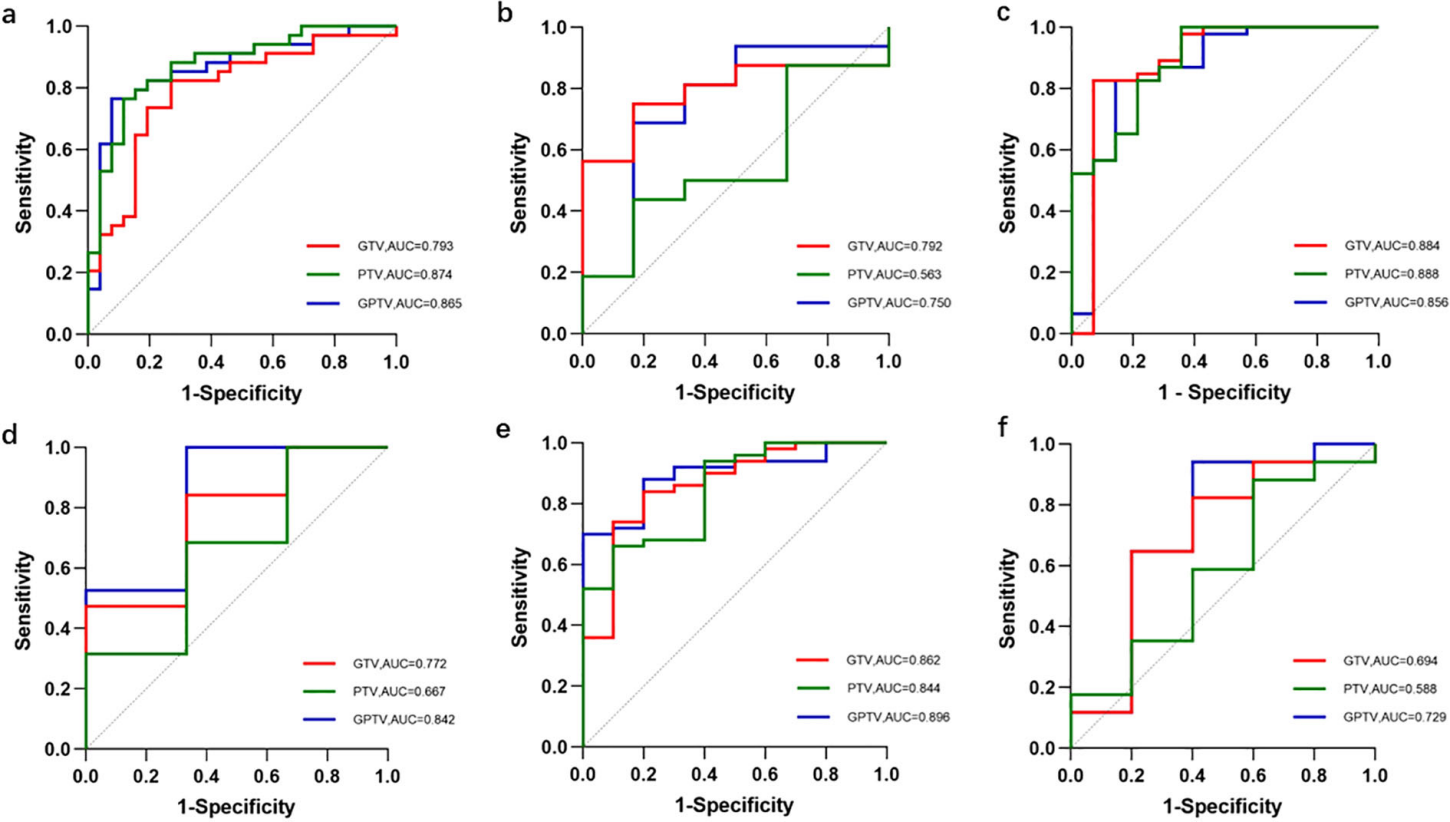
The ROC curves of the GTV, PTV, and GPTV model for each protein expression in the training and validation sets. **(a)** The ROC curves to predict PTEN expression status in the training set. **(b)** The ROC curves to predict PTEN expression status in the validation set. **(c)** The ROC curves to predict PIK3CA expression status in the training set. **(d)** The ROC curves to predict PIK3CA expression status in the validation set. **(e)** The ROC curves to predict mTOR expression status in the training set. **(f)** The ROC curves to predict mTOR expression status in the validation set.

**Figure 6:**
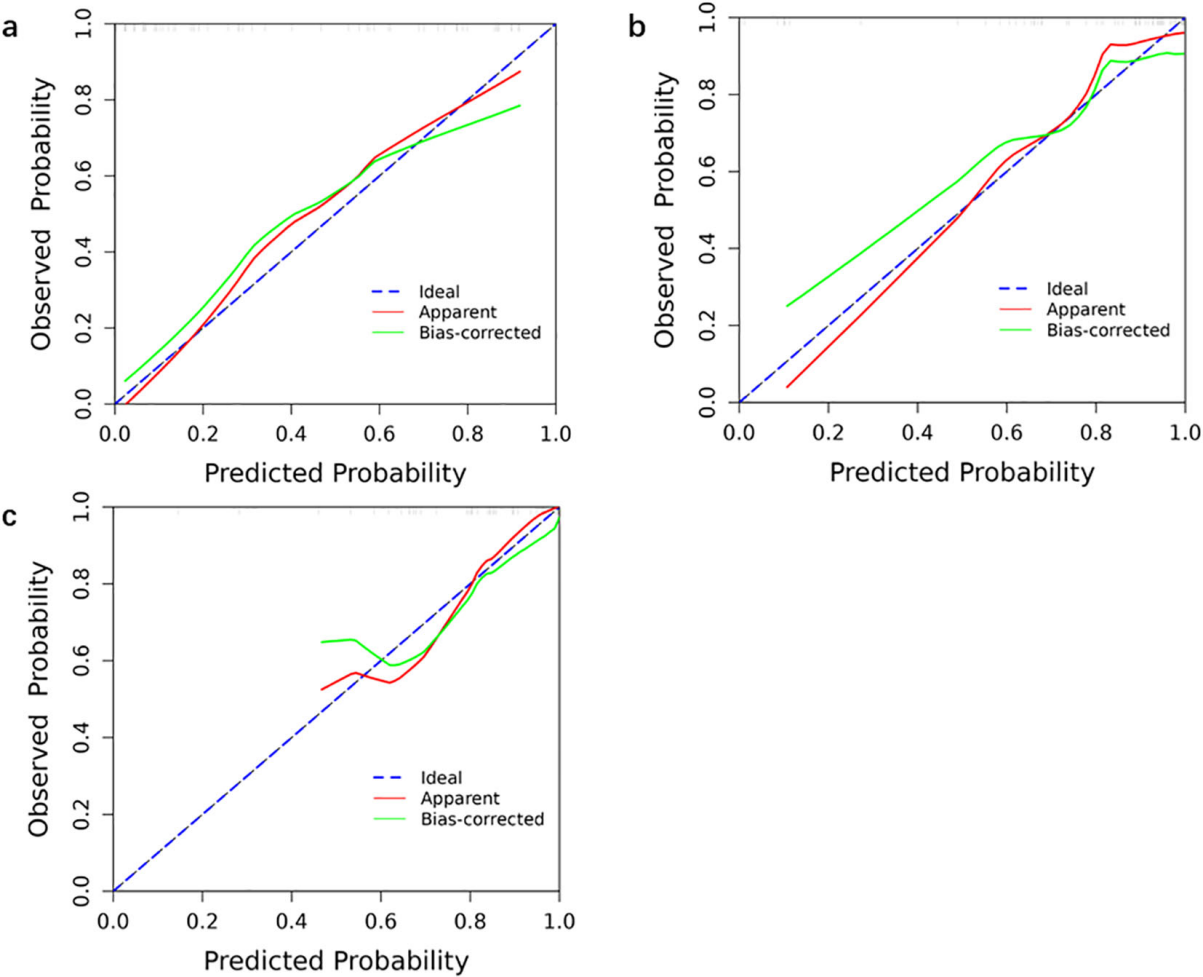
Calibration curves of the three models for each protein expression in the training. Calibration curves of the combination model that predict protein expression status in the training set **(a)** PTEN; **(b)** PIK3CA; **(c)** mTOR. The blue line is the ideal calibration curve when the predicted value is equal to the actual value. The closer the model calibration curve is to this line, the better the prediction ability of this model is.

**Figure 7:**
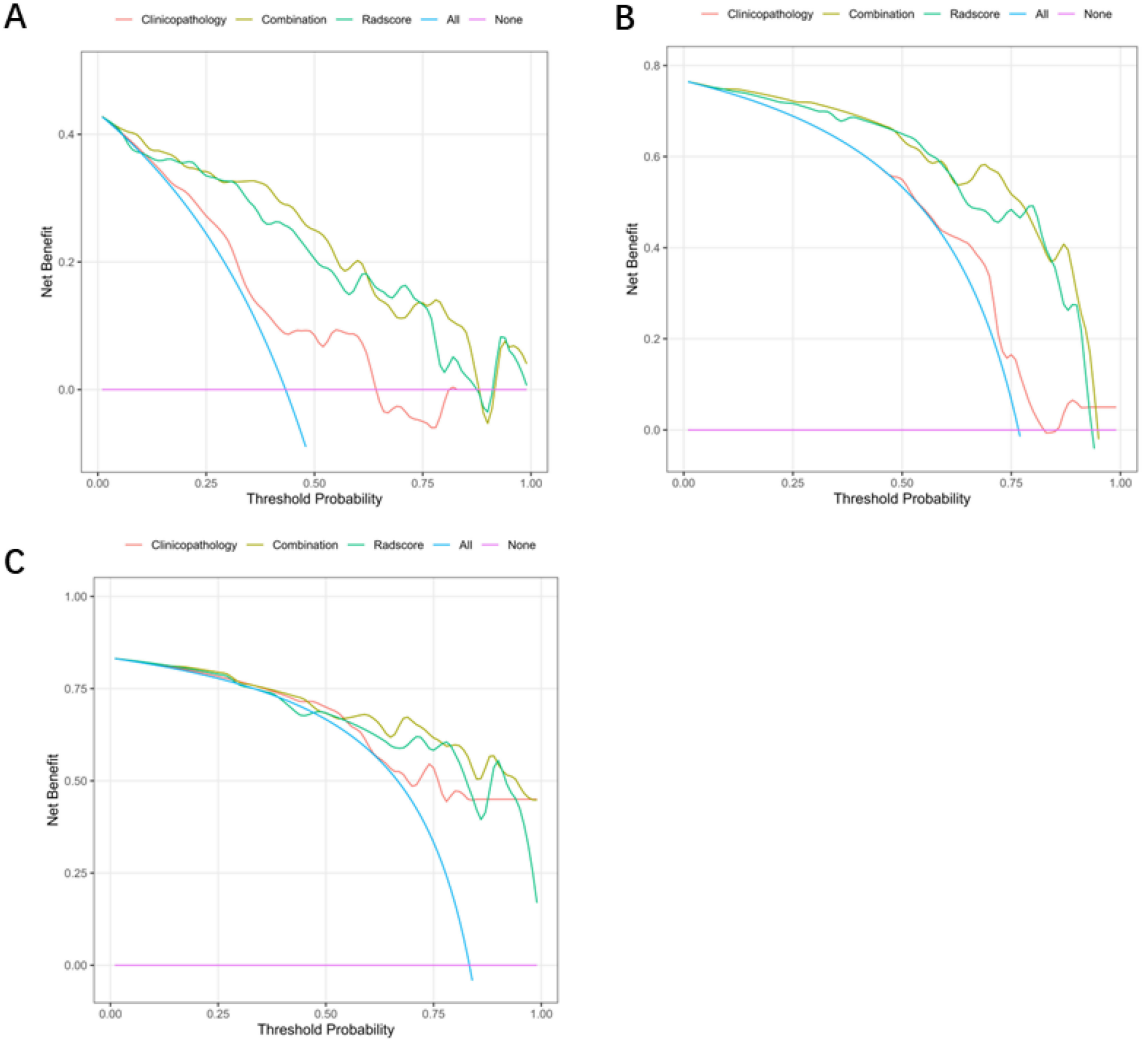
DCA of the three models for each protein expression in the training. DCA of the combination model that predict protein expression status in the training set **(A)** PTEN; **(B)** PIK3CA; **(C)** mTOR. The abscissa represents the risk of disease occurrence, the ordinate represents the patient’s net income rate, and the blue curve represents that all patients have adverse outcomes. When the prediction model curve is above the blue curve, it represents that the corresponding patients can benefit.

The ROC curves of the three models for each protein expression in the training and validation sets are shown in **Figure 4**. In the training set, the AUC values for PTEN expression prediction were 0.875, 0.703, and 0.891 for the Radscore, Clinicopathology, and Combination models, respectively. In the validation set, the corresponding AUC values were 0.750, 0.844, and 0.833 (**Figure 5a, b**). For predicting PIK3CA expression, the training set AUC values for the models were 0.856, 0.633, and 0.880, while in the validation set, they were 0.842, 0.667, and 0.825(**Figure 5c, d**). Lastly, in the training set, the AUC values for mTOR expression prediction were 0.896, 0.831, and 0.912 for the three models, respectively, with corresponding validation set AUC values of 0.729, 0.847, and 0.829 (**Figure 5e, f**). The AUC, sensitivity, specificity, and accuracy values are presented in **Table 4**. Calibration curve analysis (**Figure 6**) and decision curve analysis (**Figure 7**) showed that the joint model had good calibration and clinical utility in predicting the expression of three different proteins.

**Table 4a:** Predictive performance of PTEN expression correlation models.

Model	AUC (95% CI)	Training cohort			AUC (95% CI)	Test cohort		
		SEN	SPE	ACC		SEN	SPE	ACC
Radscore	0.865 (0.769, 0.962)	0.765	0.923	0.833	0.750 (0.500, 0.999)	0.688	0.833	0.500
Clinico-pathology	0.703 (0.571, 0.835)	0.706	0.615	0.667	0.844 (0.677, 1.000)	0.688	1.000	0.682
Combination	0.891 (0.811, 0.972)	0.824	0.885	0.850	0.833 (0.631, 1.000)	0.813	0.833	0.682

**Table 4b:** Predictive performance of PIK3-CA expression correlation models.

Model	AUC (95% CI)	Training cohort			AUC (95% CI)	Test cohort		
		SEN	SPE	ACC		SEN	SPE	ACC
Radscore	0.856 (0.719, 0.992)	0.826	0.857	0.833	0.842 (0.572, 1.000)	1.000	0.667	0.727
Clinico-pathology	0.633 (0.460, 0.805)	0.913	0.357	0.783	0.667 (0.375, 0.959)	0.790	0.667	0.727
Combination	0.880 (0.761, 1.000)	0.870	0.857	0.867	0.825 (0.589, 1.000)	0.580	1.000	0.727

**Table 4c:** Predictive performance of mTOR expression correlation models.

Model	AUC (95% CI)	Training cohort			AUC (95% CI)	Test cohort		
		SEN	SPE	ACC		SEN	SPE	ACC
Radscore	0.896 (0.810, 0.982)	0.700	1.000	0.750	0.729 (0.424, 1.000)	0.941	0.600	0.636
Clinico-pathology	0.831 (0.721, 0.941)	0.800	0.800	0.800	0.847 (0.616, 1.000)	0.882	0.800	0.818
Combination	0.912 (0.833, 0.991)	0.820	0.833	0.833	0.829 (0.600, 1.000)	0.882	0.800	0.818

AUC, the area under the curve; CI, confidence interval; SEN, sensitivity; SPE, specificity; ACC, accuracy.

In our study, we observed that the ratio of positive to negative expression of PIK3CA in the total sample was 65:17 (with a positive rate of 79.27%), and for mTOR, it was 67:15 (with a positive rate of 81.71%). This suggests that there may be class imbalance in the two types of samples, which could lead to a decrease in the specificity of the model. To avoid the accuracy paradox, this study further evaluated the recall, precision, F1-score (harmonic mean), and negative predictive value (NPV) of the models predicting the expression status of PIK3CA and mTOR, with a particular focus on the models’ ability to identify negative samples. As shown in **Table 5**, by comparing the performance metrics of the training set and the validation set, it was found that the combined model generally exhibited higher precision and F1-score in most scenarios, but its recall and negative predictive value showed some fluctuations.

**Table 5a:** The ability of the respective models to identify negative samples in predicting the expression of PIK3CA.

Model	Training cohort				Test cohort			
	Recall	Precision	F1-score	NPV	Recall	Precision	F1-score	NPV
Radscore	0.826	0.950	0.884	0.600	1.000	0.950	0.964	1.000
Clinico-pathology	0.913	0.824	0.866	0.556	0.789	0.824	0.857	0.333
Combination	0.870	0.952	0.909	0.667	0.579	0.952	0.734	0.273

**Table 5b:** The ability of the respective models to identify negative samples in predicting the expression of mTOR.

Model	Training cohort				Test cohort			
	Recall	Precision	F1-score	NPV	Recall	Precision	F1-score	NPV
**Radscore**	0.700	1.000	0.824	0.400	0.941	0.889	0.914	0.750
**Clinico-pathology**	0.800	0.952	0.869	0.444	0.882	0.938	0.909	0.667
**Combination**	0.820	0.976	0.891	0.500	0.882	0.938	0.909	0.667

The F1-Score is the harmonic mean of Precision and Recall, and its calculation formula is:F1-Score=2×(Precision× Recall)/(Precision+ Recall).

NPV, Negative Predictive Value.

**Figure 5** shows the ROC curves for the prediction of each protein expression in the training and validation sets for the gross tumor volume (GTV), peritumoral tumor volume (PTV), and combined intratumor and peritumor, i.e., Gross peritumoral tumor volume (GPTV) radiomics models. While the AUC, sensitivity, specificity, and accuracy values are presented in **Table 6**. The results showed that the performance of the GPTV model was improved over both the GTV and PTV models in predicting PIK3CA and mTOR expression. Except that the GPTV model for predicting mTOR expression showed a statistically significant difference from the GTV model in the validation set (P<0.05), the other GPTV models exhibited no statistically significant differences from the GTV model and PTV model in both the training set and the validation set (P>0.05).

**Table 6a:** Performance of GTV, PTV and GPTV models for predicting PTEN expression.

Model	AUC (95% CI)	Training cohort			AUC (95% CI)	Test cohort		
		SEN	SPE	ACC		SEN	SPE	ACC
GTV	0.793 (0.676, 0.910)	0.824	0.731	0.783	0.792 (0.602, 0.982)	0.750	0.833	0.455
PTV	0.874 (0.785, 0.964)	0.765	0.885	0.817	0.563 (0.301, 0.825)	0.438	0.833	0.500
GPTV	0.865 (0.769, 0.962)	0.765	0.923	0.833	0.750 (0.500, 0.999)	0.688	0.833	0.500

**Table 6b:** Performance of GTV, PTV and GPTV models for predicting PIK3CA expression.

Model	AUC (95% CI)	Training cohort			AUC (95% CI)	Test cohort		
		SEN	SPE	ACC		SEN	SPE	ACC
GTV	0.884 (0.747, 1.000)	0.826	0.929	0.850	0.772 (0.491, 1.000)	0.842	0.667	0.636
PTV	0.888 (0.789, 0.987)	1.000	0.643	0.917	0.667 (0.321, 1.000)	0.684	0.667	0.864
GPTV	0.856 (0.719, 0.992)	0.826	0.857	0.833	0.842 (0.572, 1.000)	1.000	0.667	0.727

**Table 6c:** Performance of GTV, PTV and GPTV models for predicting mTOR expression.

Model	AUC (95% CI)	Training cohort			AUC (95% CI)	Test cohort		
		SEN	SPE	ACC		SEN	SPE	ACC
GTV	0.862 (0.735, 0.989)	0.840	0.800	0.767	0.694 (0.397, 0.991)	0.647	0.800	0.636
PTV	0.844 (0.720, 0.968)	0.660	0.900	0.700	0.588 (0.292, 0.885)	0.882	0.400	0.455
GPTV	0.896 (0.810, 0.982)	0.700	1.000	0.750	0.729 (0.424, 1.000)	0.941	0.600	0.636

## Discussion

Targeted therapy remains an important component of precision treatment for endometrial cancer (EC) patients. Accurate prediction of the expression of molecular pathway target proteins is helpful for the development of personalized treatment plans for EC patients. This study is committed to constructing a machine learning model based on multiparametric MRI radiomics features combined with clinical pathological features, aiming to evaluate its predictive ability and clinical application value in predicting the expression status of endometrial cancer target therapy-related proteins (PTEN, PIK3CA, and mTOR). This machine learning model is expected to become a new type of non-invasive assessment tool that can accurately identify EC patients who can benefit from targeted therapy, thereby optimizing treatment decisions and significantly improving patients’ clinical outcomes.

Currently, immunohistochemistry and Western blotting are the primary methods for detecting PTEN, PIK3CA, and m-TOR expression. However, due to tumor growth heterogeneity and the limitations of local specimens in reflecting overall tumor characteristics, results from these assays can vary significantly due to technical and subjective factors. Consequently, there is a pressing clinical need for a comprehensive method to evaluate lesions, considering tumor heterogeneity, to accurately assess tumor characteristics. In comparison to conventional pelvic MRI, which is reliant on observer experience, radiomics offers a more effective approach, leveraging quantitative information extraction to capture tumor morphology, intensity, and texture features that may not be discernible to the human eye. This methodology improves the scientific rigor, objectivity, and precision of clinical diagnosis. Luo et al. (9) performed a retrospective MRI-based study and developed a column-line diagram based on clinical features and radiomic scores to assess EC lymphocyte-vascular gap invasion; Yan et al. (10) published a retrospective, multicenter study using an MRI- and Clinical-Based Radiomics Nomogram to predict preoperative high-risk endometrial cancer. The integration of radiomics and clinical features holds promise for enhancing the diagnostic efficacy of EC across various aspects such as muscle infiltration, metastasis, and risk stratification. However, there is a paucity of studies in the field of radio-proteomics. Prior research has illustrated the potential of radiomics in predicting clinically relevant molecular features and protein expression in diverse cancers, encompassing breast, lung, glioblastoma, and pancreatic cancers (11–13). In this study, we introduce a novel machine learning analysis method that amalgamates the radiomics features and clinicopathological features derived from multiparameter MRI. This approach enables an objective and comprehensive assessment of the expression status of PTEN, PIK3CA, and mTOR, offering distinct advantages over conventional pelvic MRI, immunohistochemistry (IHC), or protein blotting techniques.

PTEN stands as one of the most frequently mutated, deleted, and inactivated oncogenes in human cancers, exerting a pivotal role in the pathogenesis of EC and holding promise as a novel marker for this disease (14–16). PTEN is a tumor suppressor that causes cell cycle arrest and inhibits cell proliferation (17). Poly ADP-ribose polymerase (PARP) inhibitors are a class of targeted antitumor agents, and several experiments have confirmed the efficacy of PARP inhibitors against EC (18, 19). Notably, Emerging evidence suggests that PTEN mutant cells exhibit heightened sensitivity to PARP inhibitors due to characteristics associated with increased cellular DNA double-strand breaks and defective homologous recombination mechanisms (20, 21). It can be seen that PTEN, as a potential predictor of PARP efficacy, can provide a basis for the precise stratification and treatment of endometrial cancer patients if its expression is predicted early, thus optimizing individualized treatment and avoiding exposure to ineffective drugs. The machine learning model constructed in this study, which combines radiomics and clinical pathological features, has shown good performance in predicting PTEN expression, with a certain degree of generalizability and robustness. Ki-67, a biomolecular protein, is increasingly recognized as a preferred marker for assessing cell proliferative activity, reflecting the extent of cell growth and proliferation. Its expression holds potential as a biomarker for endometrial carcinogenesis and tumor cell proliferation (22). Previous studies have consistently reported a negative correlation between PTEN and Ki-67 expression in various malignant tumors, including head and neck tumors and lymphomas (23, 24), and a study by Uegaki K. et al. (25) demonstrated that PTEN induced cells to show cell cycle arrest and a decrease in the Ki-67 labeling index. In this study, we also confirmed through case-control analysis that there is a significant difference in the expression levels of PTEN and Ki-67 (p < 0.05), providing new experimental evidence to further elucidate the mechanism of action of PTEN in endometrial cancer.

The PIK3/AKT/mTOR signaling pathway, a classic cell signaling cascade, is among the most commonly activated pathways in malignant tumors, including EC (26). The PIK3CA gene encodes the catalytic subunit of PIK3, p110a, and exhibits frequent mutations across all molecular subtypes of endometrial cancers. mTOR, a conserved serine/threonine protein kinase, governs cell growth, survival, and migration by phosphorylating the Akt and AGC family of kinases. Consequently, aberrant activation of this pathway significantly influences tumor cell growth, proliferation, metastasis, and infiltration (27–30). Similar results were observed in this study, with a significant correlation between mTOR expression and EC pathologic grade (p < 0.05). However, due to the imbalance in baseline characteristics observed in this study (p=0.002), the subsequent validation analysis may be subject to bias. Therefore, our conclusions require further verification in larger, independent cohorts with more balanced baseline characteristics. mTOR represents a more advanced anticancer target within this signaling pathway. Notably, the reported efficacy of Everolimus (an mTOR-targeted inhibitor) in combination with letrozole (an aromatase inhibitor) for recurrent endometrial cancers (31, 32)underscores the potential for enhanced antitumor efficacy through the identification of suitable candidates for treatment with mTOR inhibitors or other targeted agents in conjunction with hormonal agents. Consequently, there is an imperative need for comprehensive characterization to optimize treatment strategies and enhance patient prognosis. The machine learning model constructed in this study, which combines radiomics and clinical pathological features, has shown good performance and high precision in predicting the expression of PIK3CA and mTOR. It outperforms both the single radiomics model and the traditional clinical pathological model in terms of calibration characteristics and clinical translation value.

In our investigation, we constructed a predictive model for the expression of targeted therapy-related proteins in endometrial cancer utilizing four sequences of CE-T1WI, FS-T2WI, DWI, and ADC images. We postulated that multiparametric imaging could more comprehensively capture the microscopic characteristics of lesions from diverse perspectives, thereby offering a more comprehensive depiction of tumor characteristics. Furthermore, this study concurrently integrated peritumoral imaging features, encompassing tumor infiltration margins, potentially shedding light on the role of the tumor microenvironment in cancer biology and behavior. Past studies across various tumor types have consistently underscored the significance of peritumoral imaging analysis in elucidating the implications for tumor biology, prognosis, and treatment response (33–35). The features employed in constructing the PTEN and mTOR machine learning models in this study all encompassed peri-tumor data, with these features carrying substantial relative weight. Additionally, we established distinct intratumoral, 3-mm peritumoral area around the tumor, and merged intratumoral and peritumoral models. Upon comparing AUC values of these models, we observed that the combined model demonstrated comparable performance to the single intratumoral model in predicting PTEN expression, whereas the combined model notably outperformed the separate models in predicting PIK3CA and mTOR expression. Furthermore, it is similar to the results of Beig et al (36), where the efficacy of distinguishing lung adenocarcinoma from sarcoidosis by combining intranodal and perinodal CT imaging histology was superior to that of the intranodal model. These results emphasize that the important contribution of peri-tumor data cannot be ignored.

This study has certain limitations that need to be further improved in subsequent research. First, the samples were only sourced from two medical centers, with a relatively limited sample size, while radiomics studies generally rely on large-scale datasets to ensure the reliability of the results. Although this study has conducted preliminary validation on an external dataset, its clinical application value still needs to be further confirmed through multicenter, prospective studies to verify the robustness, generalizability, and reproducibility of the model. Second, due to incomplete information for some patients, important clinical parameters such as weight, body mass index (BMI), estrogen levels, and CA125 could not be included in the model. In addition, under the retrospective design, the temporal heterogeneity of historical data from each center (such as the iteration of imaging equipment and the update of diagnostic criteria) may introduce inevitable confounding biases, and the differences caused by the different models of MRI equipment in the two centers cannot be ignored. Future research needs to further improve the data collection mechanism, use techniques such as resampling to mitigate potential class imbalance as much as possible, and ensure the comprehensive integration of clinical and imaging features to enhance the overall performance of the model. Finally, this study mainly focused on postoperative endometrial cancer patients, but given that targeted therapy is mainly used for patients with advanced endometrial cancer, it is urgently necessary to verify the validity of the current model in the context of advanced disease in future research.

## Conclusion

In summary, we developed a ML model based on clinicopathologic features and multi-parametric MRI-based radiomic features with strong predictive value for the expression status of PTEN, PIK3AC, mTOR. This innovative approach could be a potential tool to objectively and non-invasively provide clinical information on targeted therapy for identifying EC patients who are most likely to benefit from personalized targeted therapy.

## Data Availability

The raw data supporting the conclusions of this article will be made available by the authors, without undue reservation.
