# Correction: Development of a machine learning model for predicting the expression of proteins associated with targeted therapy in endometrial cancer

**DOI:** 10.3389/fonc.2026.1790105

**Published:** 2026-01-26

**Authors:** Chenwen Sun, Qianling Li, Yanan Huang, Yang Xia, Meiping Li, Xiucong Zhu, Jinke Zhu, Zhenhua Zhao

**Affiliations:** 1 Department of Radiology, Shaoxing People’s Hospital, Shaoxing, China; 2 School of Medicine, Graduate School, Zhejiang University, Hangzhou, China; 3 Department of Radiology, Shaoxing Maternity and Child Health Care Center, Shaoxing, China

**Keywords:** endometrial carcinoma, machine learning, Pten, PIK3, mTOR, targeted therapy

Affiliation ^1^Department of Radiology, Shaoxing People’s Hospital, Shaoxing, China was erroneously given as ^2^School of Medicine, Graduate School, Zhejiang University, Hangzhou, China for author Qianling Li.

Affiliation ^2^School of Medicine, Graduate School, Zhejiang University, Hangzhou, China.

was erroneously given as ^1^Department of Radiology, Shaoxing People’s Hospital, Shaoxing, China for author Yanan Huang, Xiucong Zhu, Jinke Zhu and Zhenhua Zhao.

The original version of this article has been updated.

